# Integrated multi-omics for potential biomarkers and molecular mechanism of persistent inflammatory refractory rheumatoid arthritis

**DOI:** 10.3389/fimmu.2025.1574783

**Published:** 2025-07-25

**Authors:** Ping-Heng Zhang, Ya-Nan Bi, Xiao-Feng Zhao, Kang Chen, En-Sheng Chen, Chang-Hong Xiao

**Affiliations:** ^1^ Rheumatology & Immunology Department, Southern Medical University Hospital of Integrated Traditional Chinese and Western Medicine, Southern Medical University, Guangzhou, China; ^2^ The Second Clinical Medical College of Guangzhou University of Chinese Medicine, Guangzhou, China; ^3^ State Key Laboratory for Quality Ensurance and Sustainable Use of Dao-di Herbs, National Resource Center for Chinese Materia Medica, China Academy of Chinese Medical Sciences, Beijng, China

**Keywords:** persistent inflammatory refractory rheumatoid arthritis, multi-omics, golgi apparatus, tryptophan metabolism, lipid metabolism

## Abstract

**Introduction:**

Persistent inflammatory refractory rheumatoid arthritis (PIRRA) presents a major clinical challenge, and its underlying molecular mechanisms remain inadequately understood.

**Methods:**

athogenesis. Synovial joint tissues were collected from 30 TgTC mice and 30 Friend virus B (FVB) control mice. Of these, 18 mice per group were used for transcriptomic, proteomic, and metabolomic analyses; 6 for pathological examination and microCT imaging; and 6 for validation experiments. Gene Ontology (GO), Kyoto Encyclopedia of Genes and Genomes (KEGG) pathway analysis, protein-protein interaction networks, and KEGG Markup Language (KGML) network analysis were employed to characterize the functional roles of differentially expressed genes (DEGs), proteins, metabolites, and associated biological pathways. Notably, five genes/proteins—macrophage-expressed gene 1 (*Mpeg1*), ectonucleotide pyrophosphatase/phosphodiesterase 2 (*Enpp2*), toll-like receptor 2 (*Tlr2*), cluster of differentiation 14 (*CD14*), and lysozyme 2 (*Lyz2*)—were validated by quantitative reverse transcription PCR (qRT-PCR), Western blotting, and immunohistochemistry.

**Results:**

A total of 2,410 DEGs, 366 differentially expressed proteins, and 120 significantly altered metabolites (P < 0.05) were identified between the model (TgTC ) and control (FVB) groups. These molecules were mainly associated with Golgi apparatus dysfunction, lipid metabolism, and immune-inflammatory responses. Integrative multi-omics analysis further revealed that these molecular alterations are involved in the activation of the PI3K-AKT-mTOR signaling pathway, as well as disruptions in tryptophan and lipid metabolism. Among the metabolites, phosphatidylinositol (PI) (12:0/12:0), N-docosahexaenoyl tryptophan, and PI (22:1(11Z)/0:0) were identified as key metabolic signatures of persistent joint synovitis in TgTC mice. In addition, the expression of *Mpeg1, Enpp2, Tlr2, CD14*, and *Lyz2* was evaluated in synovial samples from patients with PIRRA and classical RA. Notably, *Mpeg1, Enpp2*, and *Lyz2* were significantly upregulated in PIRRA, whereas *Tlr2* and *CD14* did not show statistically significant differences between groups.

**Discussion:**

Our findings highlight the critical role of altered gene, protein, and metabolite expression in the pathogenesis of PIRRA, offering new insights into its molecular basis and potential therapeutic targets.

## 1 Introduction

Persistent inflammatory refractory rheumatoid arthritis (PIRRA) is a relatively rare and difficult-to-treat subtype of rheumatoid arthritis (RA) (1). In clinical practice, despite appropriate and continuous drug treatment, PIRRA patients continue to experience persistent arthritis, uncontrolled disease activity, and progressive joint injury, leading to severe disability, accelerated atherosclerosis, tumor development, and early death (2). Consequently, PIRRA profoundly affects the quality of life of patients, bringing heavy burdens to their families and society.

The etiology and pathogenesis of PIRRA remain unclear, and current research on its pathogenesis is limited. Some recent studies have begun to explore potential contributing factors. For instance, Rogers et al. reported that RA patients with a significant history of smoking are more likely to develop PIRRA (3). Van et al. observed that RA patients with positive anti-cyclic citrullinated peptide exhibit more pronounced synovial tissue infiltration and prolonged duration of inflammation (4). Savola et al. proposed that somatic mutations may contribute to the sustained proliferation of synovial cells, thereby perpetuating joint inflammation and potentially linking PIRRA to oncogenic processes (5). Similarly, Stylianou et al. suggested that epigenetic reprogramming, including DNA methylation and histone modification, plays a key regulatory role in chronic inflammatory diseases and may be involved in the pathogenesis of PIRRA (6). These findings suggest possible associations between PIRRA and factors such as smoking, autoantibodies, somatic mutations, and epigenetic changes. However, these studies remain largely exploratory, and comprehensive investigations are needed to determine the specific molecular mechanisms underlying PIRRA.

With the advancement of omics technologies, multi-omics methods have been successfully applied to the diagnosis, treatment, and prognosis of various diseases (7), especially cancer (8). However, to date, no studies have reported the application of multi-omics analyses to PIRRA. Addressing this gap, multi-omics investigations hold promise for deepening our understanding of the underlying mechanisms of PIRRA. Due to the challenges associated with obtaining synovial tissue samples from patients, we employed a human tumor necrosis factor-alpha (TNF-α) transgenic (TgTC) mouse model of arthritis as an experimental surrogate. The TgTC mouse model develops spontaneous arthritis, and its joint pathology closely mirrors that observed in PIRRA, both in terms of histopathological features and clinical manifestations. In this study, we utilized TgTC mice as the experimental model to perform a comprehensive multi-omics analysis—encompassing transcriptomics, proteomics, and metabolomics—of synovial tissues from arthritic TgTC mice and healthy control mice. The study aimed to identify potential biomarkers and elucidate molecular mechanisms relevant to PIRRA, thereby providing foundational evidence and novel targets to support future clinical diagnosis and therapeutic strategies.

## 2 Materials and methods

### 2.1 The rationale for selecting the TgTC mouse arthritis model

Given the challenges in obtaining synovial tissue samples from patients, a human TgTC mouse model of arthritis was selected as an alternative for studying PIRRA. The rationale for this choice is based on the following considerations:

1. PIRRA is a distinct and difficult-to-treat subtype of RA, characterized by a complex pathogenesis involving persistent synovial inflammation, tumor-like synovial fibroblast (SF) proliferation, progressive bone erosion, and resistance to multiple therapeutic agents. Currently, no animal model fully replicates the human condition. Traditional models such as collagen-induced arthritis (CIA) and adjuvant-induced arthritis (AIA) exhibit self-limiting inflammation and lack the pronounced synovial hyperplasia characteristic of late-stage PIRRA. In contrast, the TgTC mouse model spontaneously develops chronic arthritis. Histopathological analysis reveals extensive tumor-like proliferation of SFs, significant inflammatory cell infiltration, cartilage degradation, and bone erosion. Moreover, SFs isolated from TgTC mice and transplanted into healthy mice induce sustained synovitis, cartilage destruction, and bone erosion—closely reflecting the clinical and pathological features of PIRRA. Unlike acute inflammation models, the TgTC model demonstrates chronic and progressive joint inflammation, aligning more closely with the disease course of PIRRA (9, 10).
2. Clinically, PIRRA is notable for its poor responsiveness to a broad range of pharmacologic therapies. This feature is mirrored in the TgTC mouse model. Despite being a TNF-α transgenic model, TgTC mice do not exhibit a full therapeutic response to TNFα antagonists (11). Furthermore, studies indicate that the development of arthritis in TgTC mice is not only associated with the TNF-α signaling pathway but also with other genetic modifications and molecular mechanisms. For example, there is an upregulation of SUMO-activating enzyme SEA1/UBA2 in TgTC mice, which mediates PKM2 phosphorylation, promotes SF glycolysis, and contributes to the progression of arthritis (12). Moreover, TgTC mice display impaired immune tolerance in both T cells and B cells, resulting in excessive production of inflammatory cytokines and deposition of immune complexes within the joint cavity. These events subsequently exacerbate joint inflammation and bone erosion (13). Therefore, the TgTC model effectively recapitulates the reduced responsiveness of PIRRA to conventional immunomodulatory therapies.
3. In both RA and refractory RA (RRA), TNF-α is a central pro-inflammatory cytokine positioned at the top of the inflammatory cascade. It drives the tumor-like proliferation of SFs and promotes the release of inflammatory mediators and matrix metalloproteinases, resulting in chronic joint inflammation, progressive cartilage breakdown, and bone destruction. Recent whole-genome sequencing studies have shown that chronic TNF-α exposure can induce a stable, inflammatory, and metabolically active phenotype in rheumatoid arthritis synovial fibroblasts (RA-SFs). These findings underscore the pivotal role of TNF-α signaling in the pathogenesis of PIRRA and further validate the TgTC mouse model as a relevant system for its study (14).

### 2.2 Animal experiments

Thirty female Friend virus B (FVB) mice (15–20 g, 6 weeks old) and 30 TgTC mice (15–20g, 6 weeks old) were purchased from the Guangdong Experimental Animal Monitoring Institute (SCXK (Yue) 2018-0044). The mice were randomly assigned into two groups: the normal (NC) group (30 FVB mice) and the model (MC) group (30 TgTC mice). Mice from both groups were co-housed in individual cages (maximum of six mice per cage) to ensure identical environmental conditions throughout the study. All mice were housed in a specific pathogen-free (SPF) environment at the Laboratory Animal Center of Southern Medical University under controlled conditions: temperature of 20–25 °C, relative humidity of 50–65%, and a 12-hour light/dark cycle. Food and water were provided ad libitum. Following a 3-day acclimatization period, the mice were used in the experimental procedures. Of the total, 18 FVB and 18 TgTC mice were allocated for transcriptomic, proteomic, and metabolomic analyses; 6 FVB and 6 TgTC mice were used for histopathological assessments and micro-computed tomography (micro-CT) imaging; and the remaining 6 FVB and 6 TgTC mice were reserved for follow-up validation experiments. Throughout the 15-day experimental period, body weight, paw thickness, and arthritis scores were recorded every three days. At the end of the experiment, the 8-week-old mice were anesthetized via intramuscular injection of 50 mg/kg Shu Tai, followed by euthanasia through cervical dislocation. To sterilize the surface, specimens were immersed in 75% ethanol for three minutes. The skin overlying the joints (hip, knee, and ankle) was incised, and the underlying musculature was carefully dissected using glass instruments. Synovial tissues were precisely excised from the joint capsule margins using microscissors and forceps, with constant hydration maintained using physiological saline. Immediately following excision, blood contamination was removed by rinsing the tissues with pre-cooled RNase-free phosphate-buffered saline (PBS). Excess fluid was gently blotted, and tissues were transferred into pre-chilled, enzyme-free tubes. Samples were then snap-frozen in liquid nitrogen and stored for subsequent transcriptomic, proteomic, and metabolomic sequencing analyses.

### 2.3 Histopathological examinations

After 8 weeks of decalcification with ethylenediaminetetraacetic acid (EDTA), the right ankle joints from 6 mice in each group were harvested, fixed in formalin for 12 hours, dehydrated, embedded in paraffin, and sectioned into 5 μm slices. The tissue sections were then stained with hematoxylin and eosin (H&E), safranin O, and toluidine blue.

### 2.4 MircoCT

The left ankle joint and whole paw of 3 mice from each group were fixed in a 4% paraformaldehyde solution for 24 hours, after which the specimens were mounted on a MicroCT stage for scanning. Scanning was performed using a radiation tube set to 200 μA current and 70 kV voltage, with a resolution of 6.534 μm, an exposure time of 350 ms, and a scanning angle of 180 degrees. The region of interest (ROI) was defined and analyzed, and a 3D image was generated for subsequent software-based analysis. The primary detection parameters included bone volume fraction (BV/TV), trabecular separation (Tb. Sp), trabecular pattern factor (Tb. Pf), and trabecular thickness (Tb. Th).

### 2.5 Quantitative reverse transcription PCR

Total RNA was extracted from the joint synovial tissues of 6 mice from each group using the TRIzol reagent, following the manufacturer’s instructions. The extracted RNA sample was reverse transcribed into cDNA using a reverse transcription kit. cDNA amplification was performed with the SYBR Green PCR kit. The PCR reaction setup was as follows: 5 μL SYBR Green qPCR Master Mix, 1 μL cDNA template, 0.5 μL of each upstream and downstream primer, and 3 μL of ddH_2_O. The amplification conditions were: initial denaturation at 95°C for 3 minutes, followed by 40 cycles of denaturation at 95°C for 15 seconds, and annealing at 60°C for 30 seconds. β-actin was used as an internal reference gene, and the relative expression levels were calculated using the 2^-ΔΔCt^ method.

### 2.6 Western blotting

Proteins were extracted from the joint synovial tissues of 3 mice per group using RIPA lysis buffer. Protein concentrations were determined using a bicinchoninic acid (BCA) assay kit. The total protein was then subjected to electrophoresis, membrane transfer, and blocking. After washing, the membranes were incubated overnight at 4 °C with the following primary antibodies: pyrophosphatase/phosphodiesterase 2 (*Enpp2*; 1:1000), toll-like receptor 2 (*Tlr2*; 1:500), cluster of differentiation 14 (*CD14*; 1:500), lysozyme (*Lyz2*; 1:1000), phosphorylated phosphoinositide 3-kinase*p85(Tyr467)(p-PI3Kp85(Tyr467)*; 1:1000), phosphorylated protein kinase B*(Ser473) (p-AKT(Ser473)*; 1:1000), phosphorylated mammalian target of rapamycin *(Ser2481)(p-mTOR(Ser2481)*; 1:500), glyceraldehyde-3-phosphate dehydrogenase (*GAPDH*; 1:5000), and *β-actin* (1:10000). The membranes were then washed three times with Tris-buffered saline containing 0.1% Tween-20 (TBST) for 15 minutes each. Horseradish peroxidase (HRP)-conjugated secondary antibodies (1:10000) were added, and the membranes were incubated on a shaker at room temperature for 2 hours. Following incubation, the membranes were washed again three times with TBST for 15 minutes each. Protein bands were visualized using an enhanced chemiluminescence (ECL) Plus detection kit, exposed, and imaged. *β-actin or GAPDH* was used as an internal reference protein, and the absorbance values of each protein were quantified using ImageJ software. The relative expression levels of target proteins were calculated as the ratio of target protein to *β-actin or GAPDH*.

### 2.7 Immunohistochemistry

The right ankle joints of 3 mice per group were embedded in paraffin and sectioned into 5 μm slices. After deparaffinization using a graded ethanol series, antigen retrieval was performed using an appropriate antigen-repair solution. The sections were then washed with PBS and incubated overnight at 4°C with the following primary antibodies: *Mpeg1* (1:200), *Enpp2* (1:200), *Lyz2* (1:200), *Tlr2* (1:200), and *CD14* (1:200). Following primary antibody incubation, the sections were washed with PBS and incubated with a secondary antibody (1:1000) at room temperature for 40 minutes. Color development was carried out using 3,3′-diaminobenzidine (DAB), followed by counterstaining with hematoxylin. The sections were then rinsed with running water, dehydrated through a graded ethanol series, and mounted with a coverslip. The sections were observed under a light microscope, and the expression of target proteins was quantitatively analyzed using Image-Pro Plus 6.0 software.

### 2.8 Transcriptome analysis

Transcriptome analysis was performed on six biological replicate samples from each group using RNA sequencing. Total RNA was extracted using a commercial RNA extraction kit, and RNA integrity (RIN) was assessed using the Agilent 2100 Bioanalyzer (Agilent Technologies, Santa Clara, CA, USA). Transcriptome libraries were constructed following the manufacturer’s instructions using the VAHTS Universal V6 RNA-seq Library Prep kit. RNA sequencing and data analysis were outsourced to OE Biotech, Inc. (Shanghai, China).

Sequencing was performed on the Illumina NovaSeq 6000 platform, generating 150 bp paired-end reads. Raw sequencing reads were processed using FastP to remove adapters and low-quality sequences. Clean reads were aligned to the mouse reference genome using HISAT2. Gene expression levels were quantified as fragments per kilobase of transcript per million mapped reads (FPKM), and raw read counts for each gene were obtained using HTSeq-count. Principal component analysis (PCA) was conducted using R (v3.2.0) to evaluate the biological replicates. Differentially expressed genes (DEGs) were identified using the DESeq2 software, with selection criteria of q-value < 0.05 and |log2FoldChange| > 1. Identified DEGs were subjected to Gene Ontology (GO) and Kyoto Encyclopedia of Genes and Genomes (KEGG) pathway enrichment analyses to explore associated biological functions and pathways.

### 2.9 Proteomics analysis

Total protein was extracted from joint synovial tissues from each group, and protein concentration was determined using the BCA method. The protein solutions were then stored at -80°C for subsequent analysis. Proteomic sequencing was outsourced to OE Biotech, Inc. (Shanghai, China). Following the quality assessment of the protein samples, enzymatic digestion was performed using trypsin, and the resulting peptides were labeled with tandem mass tag (TMT, also known as iTRAQ) reagents. After incubation at room temperature for 1 hour, the reaction was terminated by the addition of 5% hydroxylamine. The samples were then freeze-dried and stored at -80°C.

Sample components were initially separated using high-performance liquid chromatography (HPLC; Agilent 1100 system) equipped with an Agilent Zorbax Extend C18 column (2.1 × 150 mm, 5 μm). The resulting fractions were further separated using an EASY-nLC 1200 liquid chromatography system (Thermo Fisher Scientific) at a flow rate of 300 nL/min. Peptides were subsequently analyzed using a Q Exactive HF mass spectrometer (Thermo Fisher Scientific) following ultra-high-performance liquid chromatography (UHPLC) separation. Differentially expressed proteins (DEPs) were identified based on fold changes in sample expression levels (Foldchange ≥ 1.5 or Foldchange ≤ 1/1.5) and *P*-value < 0.05. Identified DEPs were then subjected to GO functional clustering and KEGG pathway enrichment analysis. For protein interaction analysis, the STRING database (https://string-db.org/) was used to retrieve interaction data for DEPs, selecting the relevant species (with a blast e-value of 1e-5). The top 25 proteins with the highest connectivity rankings were selected, and their interaction data were used to construct network and bar plots illustrating the protein interaction landscape.

### 2.10 Untargeted LC-MS metabolomics analysis

Metabolomic sequencing and analysis were performed by Luming Biotechnology Co., Ltd. (Shanghai, China). Metabolic profiling was performed using a liquid chromatography-mass spectrometry (LC-MS) system consisting of an ACQUITY UPLC I-Class Plus ultra-high-performance liquid chromatography system coupled with a QE Plus high-resolution mass spectrometer (Thermo Fisher Scientific, Waltham, MA, USA), equipped with an electrospray ionization (ESI) source. Separation was achieved using an ACQUITY UPLC HSS T3 column (1.8 μm, 2.1 × 100 mm), operating under both positive and negative ionization modes. The mass spectrometry scan range was set from 100 m/z to 1200 m/z, with a resolution of 70,000 for full MS and 17,500 for MS/MS. Collision energies were set at 10, 20, and 40 eV.

Raw LC-MS data were processed using Progenesis QI V2.3 (Nonlinear Dynamics, Newcastle, UK) for baseline filtering, peak identification, integration, retention time correction, peak alignment, and normalization. The data matrix was imported into the R package for PCA, which was used to assess the overall distribution of samples and the stability of the analysis process. Orthogonal partial least squares discriminant analysis (OPLS-DA) was employed to identify metabolite differences between the groups. Differentially expressed metabolites (DEMs) between the normal control (NC) and TgTC model groups were identified using Student’s *t*-test. Metabolites with variable importance in projection (VIP) score > 1.0 and *P*-value < 0.05 were defined as DEMs.

### 2.11 Synovial tissue samples

Synovial tissue samples were obtained from the knee joints of three patients diagnosed with RA and three patients diagnosed with PIRRA. All RA cases met the 2010 diagnostic criteria established by the American College of Rheumatology/European League Against Rheumatism (ACR/EULAR), while PIRRA cases fulfilled the 2021 EULAR criteria for RRA. Each patient underwent a comprehensive clinical assessment, including detailed physical examination and history taking, conducted by two independent rheumatology specialists. The diagnosis of PIRRA was established only upon full consensus between the evaluating experts. A diagnosis of PIRRA was confirmed only when there was full consensus among all experts. The synovial tissues were excised under minimally invasive needle knife arthroscopy, fixed in 4% formaldehyde, and embedded for subsequent IHC analysis.

### 2.12 Statistical analysis

Statistical analyses were conducted using Linux (Ubuntu 20.04.6), Python (version 3.7.9), R (version 4.2.3), and SPSS (version 22.0). For comparisons between two groups, the Wilcoxon rank-sum test was employed when the data did not meet the assumptions of normality. When the data satisfied the conditions for parametric analysis, independent sample t-tests were used. Spearman correlation analysis was performed to assess associations between species and metabolites. P<0.05 was considered statistically significant.

## 3 Results

### 3.1 TNF-α induced joint inflammation and bone destruction in mice

**Figure 1:**
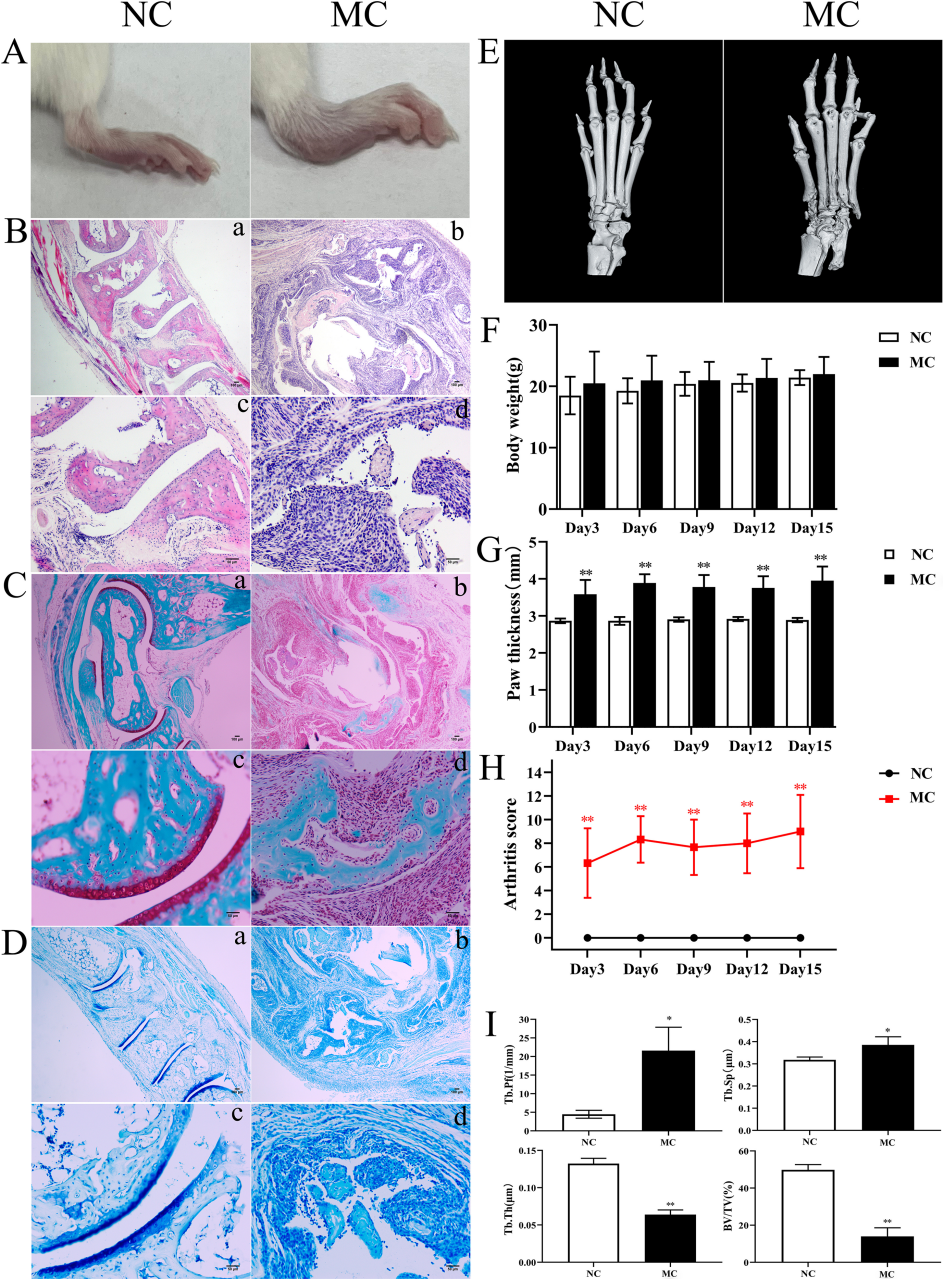
TNF-α induced joint inflammation and bone destruction. **(A)**: Joint swelling in mice. **(B)**: H&E staining. **(C)**: Safranine solid green staining. **(D)**: Toluidine blue staining. **(E)**: Representative microCT images. **(F)**: Body weight. **(G)**: Paw thickness. **(H)**: Arthritis score. **(I)**: Bone analysis parameters. ^*^ *P* < 0.05,^**^ *P* < 0.01.

To investigate the pathological features of joint synovial inflammation resembling PIRRA, a TgTC mouse model was used in this study. Body weight and paw thickness were recorded every 3 days over a 15-day period for both the normal control (NC) and model control (MC) groups. No significant difference in body weight was observed between the two groups (*P* > 0.05). However, paw thickness and arthritis scores were significantly elevated in the MC group compared to the NC group (*P* < 0.01) (**Figures 1A, F–H**). Pathological changes in the joint synovium were observed using H&E staining, safranin O/green staining, toluidine blue staining, and microCT. The MC group exhibited significant signs of joint inflammation, including extensive infiltration of inflammatory cells, synovial tissue proliferation, and protrusion of synovial tissue into the joint cavity. Degenerated and detached synovial tissue fragments were observed within the joint space, and the synovial lining was markedly thickened and multilayered. The articular surface appeared irregular, with evidence of cartilage erosion or loss. Inflammatory infiltration extended into the cartilage, accompanied by noticeable subchondral bone resorption. In contrast, the NC group exhibited a well-preserved synovial architecture, with no signs of synovial hyperplasia or protrusion, and no evidence of inflammatory cell infiltration. Synovial cells presented with distinct boundaries and minimal layering. The articular surface, cartilage, subchondral bone, and surrounding structures remained intact and free of pathological changes (**Figures 1B–E, I**).

### 3.2 Transcriptomics analysis of TNF-α transgenic mice

**Figure 2:**
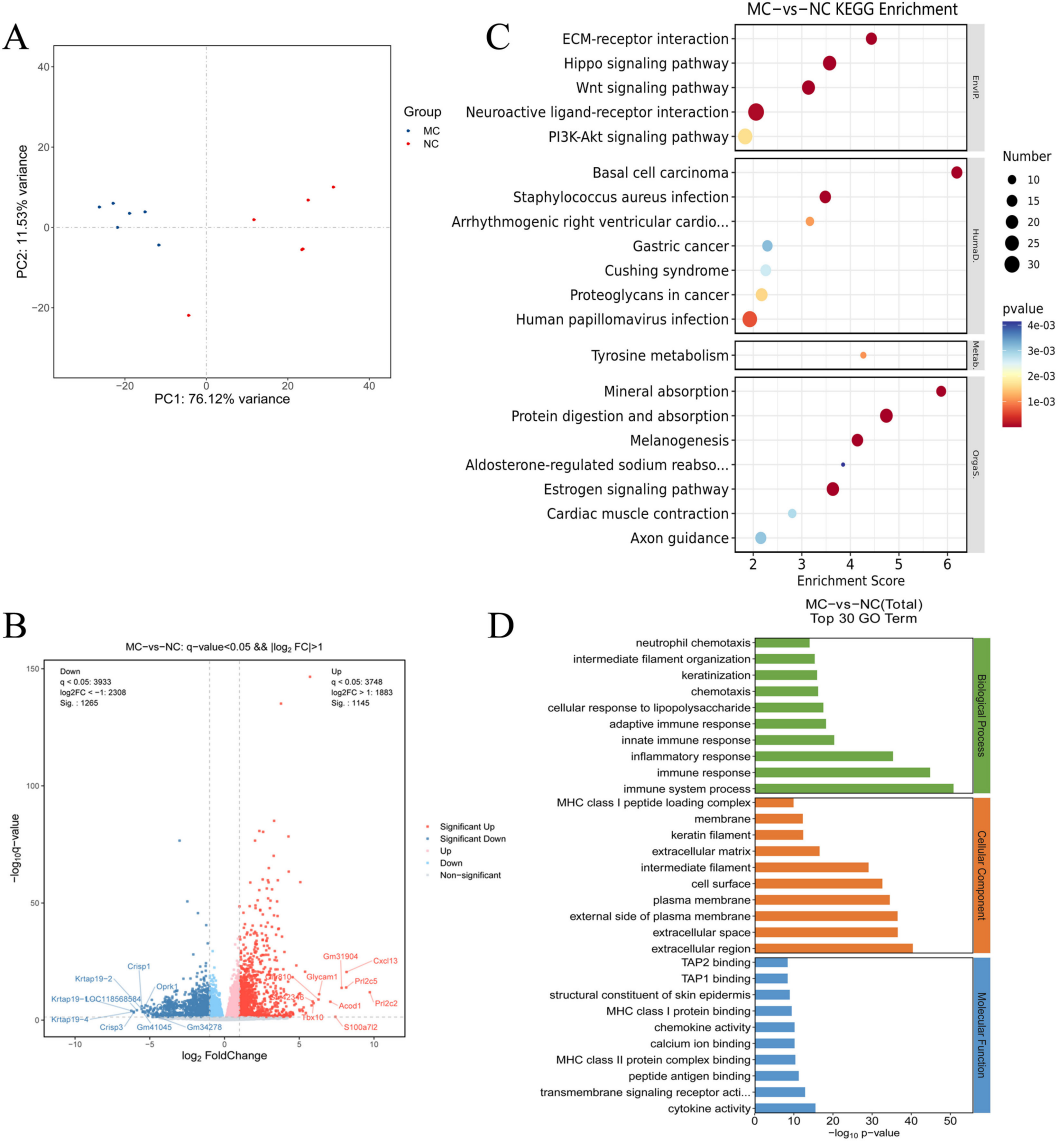
Transcriptomics analysis. **(A)**: Principal component analysis of joint synovial tissue samples in NC group and MC group. **(B)**: Volcano map analysis of differentially expressed genes in joint synovial tissues in the NC and MC groups. The gray plots indicate the non-significant genes, while the red and blue plots indicate upregulated and downregulated genes, respectively. **(C)** KEGG pathway analysis and **(D)** GO analysis of the top 30 terms associated with the DEGs.

To investigate the potential molecular mechanisms underlying PIRRA, transcriptomic analysis was conducted on synovial tissues from the joints of TgTC mice using high-throughput RNA sequencing. PCA revealed significant differences in transcript expression between TgTC and NC mice (**Figure 2A**). A total of 2,410 DEGs were identified based on the criteria of q-value < 0.05 and |log2FoldChange| > 1.0. Among these, 1,145 genes were upregulated, while 1,265 genes were downregulated (**Figure 2B**).

GO analysis revealed that the DEGs were predominantly associated with biological processes such as immune response, inflammatory response, innate and adaptive immune responses, cellular response to lipopolysaccharide, chemotaxis, and neutrophil chemotaxis (**Figure 2D**). Kyoto Encyclopedia of Genes and Genomes (KEGG) pathway analysis further demonstrated that the DEGs were significantly enriched in several signaling pathways, including extracellular matrix (ECM)-receptor interaction, Hippo signaling, Wnt signaling, neuroactive ligand-receptor interaction, PI3K-AKT signaling, estrogen signaling, melanogenesis, protein digestion and absorption, and mineral absorption (**Figure 2C**). These findings suggest that multiple signaling pathways are activated in TgTC mice. Additionally, gene set enrichment analysis (GSEA) revealed significant enrichment of PI3K-AKT signaling among the upregulated gene sets in the TgTC group compared to the NC group (**Supplementary Materials**).

### 3.3 Proteomic analysis of TNF-α transgenic mice

**Figure 3:**
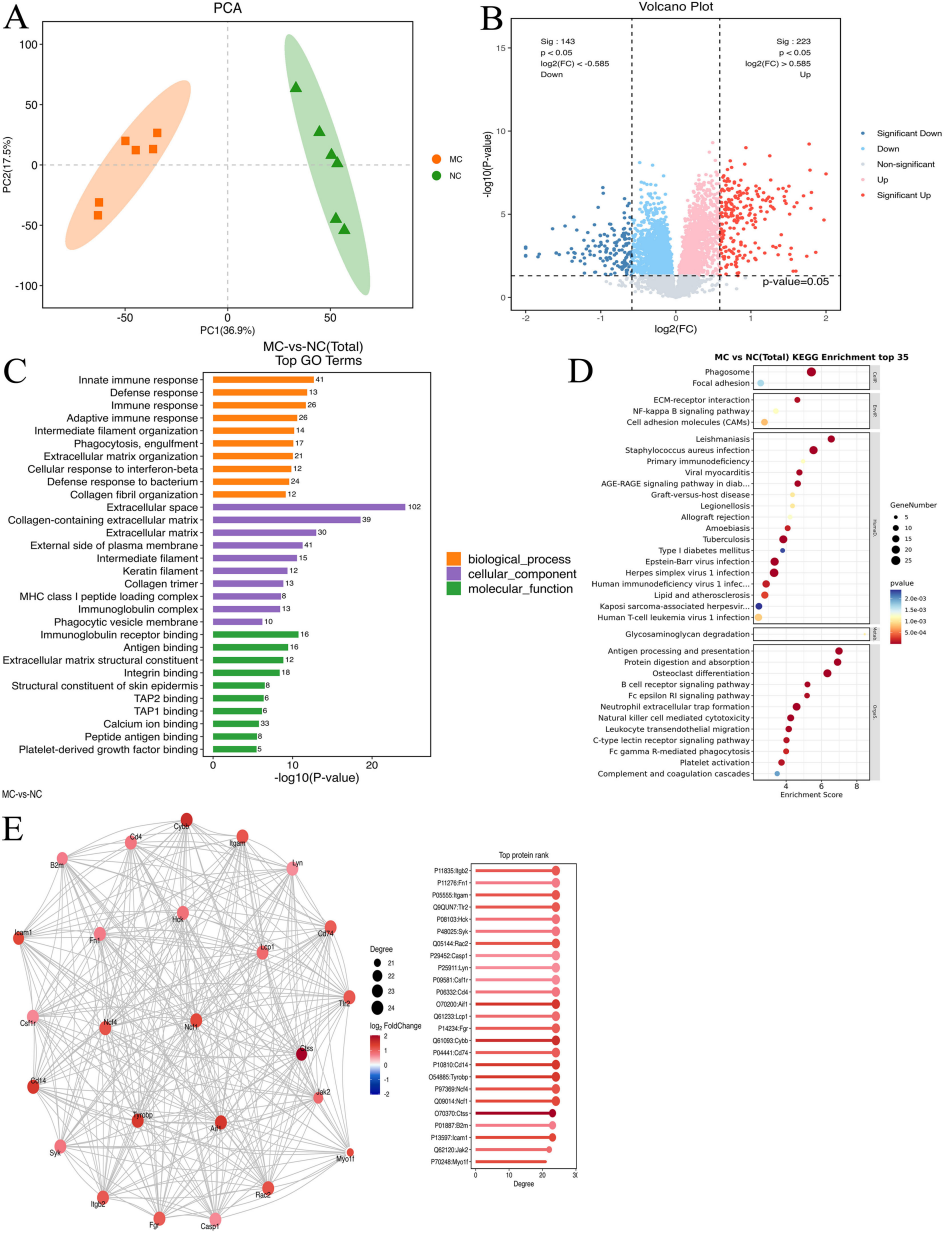
Proteomic analysis. **(A)**: Principal component analysis of joint synovial tissue samples in the NC and MC groups. **(B)**: Volcano map analysis of differentially expressed genes in joint synovial tissues in the NC and MC groups. The gray plots indicate non-significant genes, while the red and blue plots indicate upregulated and downregulated genes, respectively. **(C)** GO analysis and **(D)** KEGG pathway analysis based on the differentially expressed proteins identified in the NC and MC groups. E: The top 25 connectivity protein interaction network diagram. Circles represent differential proteins, red represents upregulation, and larger circles indicate higher connectivity.

To examine the changes in protein expression in the synovial tissues of NC and TgTC mice, tandem mass tag (TMT)-based proteomic analysis was performed on protein extracts from joint synovial tissues. PCA demonstrated a significant difference in protein abundance between the NC and MC groups (**Figure 3A**). A total of 366 DEPs were identified based on the criteria of fold change ≥ 1.5 or ≤ 1/1.5 and *P* < 0.05, of which 223 were upregulated and 143 were downregulated (**Figure 3B**).

GO enrichment analysis revealed that these DEPs were predominantly involved in immune-related biological processes, including immune response, defense response, adaptive immune response, phagocytosis, engulfment, extracellular matrix organization, cellular response to interferon-β, and collagen fibril organization (**Figure 3C**). KEGG pathway enrichment analysis further showed enrichment in pathways such as protein digestion and absorption, *Staphylococcus aureus* infection, ECM–receptor interaction, estrogen signaling, cardiac muscle contraction, focal adhesion, relaxin signaling, and PI3K-AKT signaling (**Figure 3D**). Additionally, protein-protein interaction analysis using the STRING database identified the top 25 DEPs with the highest degree of network connectivity. These included *Myo1f, Jak2, Icam1, B2m, Ctss, Ncf1, Ncf4, Tyrobp, CD14, CD74, Cybb, Fgr, Lcp1, Aif1, CD4, Csf1r, Lyn, Casp1, Rac2, Syk, Hck, Tlr2, Itgam, Fn1, and Itgb2.* Many of these proteins are associated with immune and inflammatory processes, suggesting their potential involvement in the pathogenesis of PIRRA (**Figure 3E**).

### 3.4 Integration of transcriptome and proteome datasets

**Figure 4:**
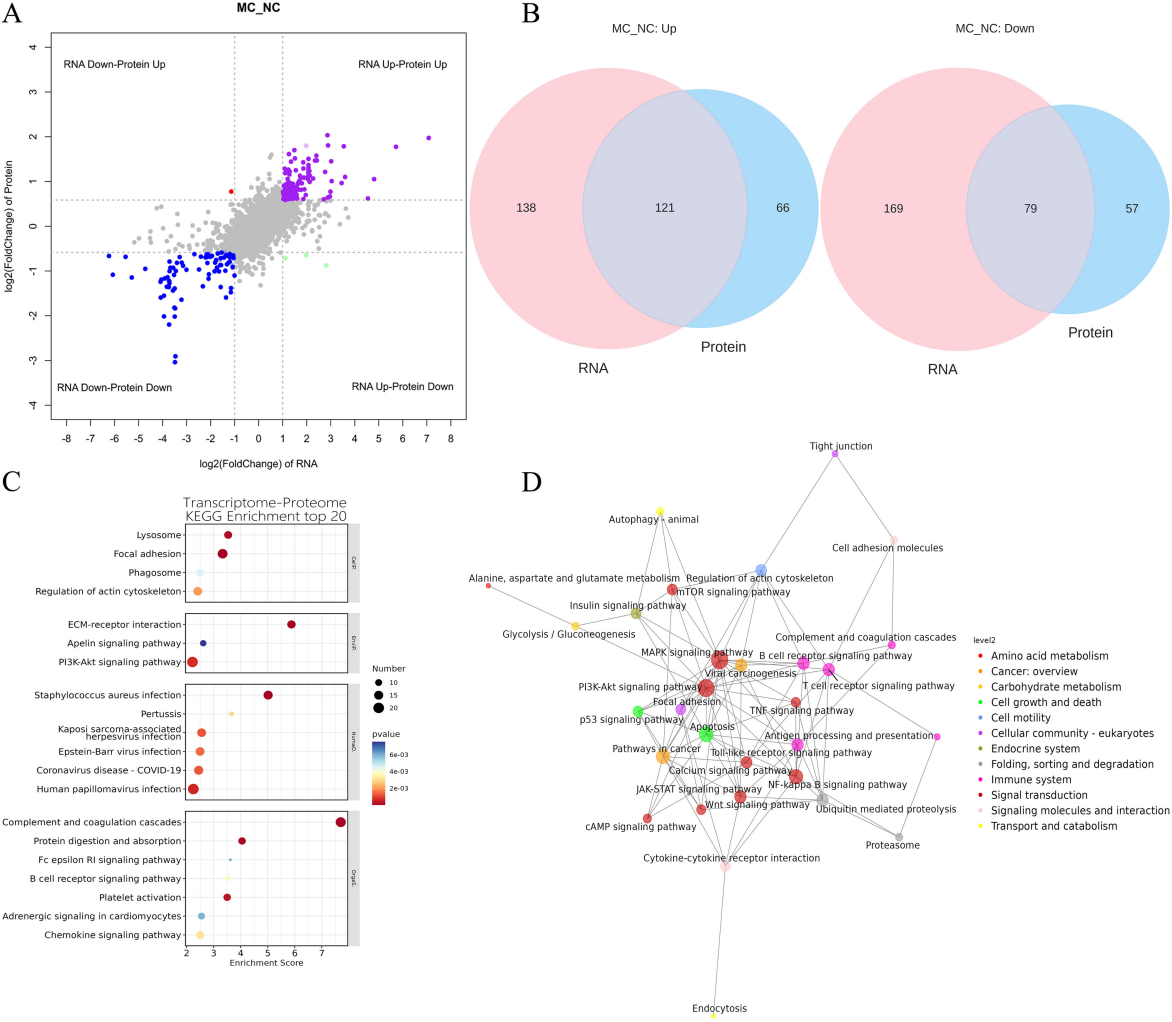
Integration of transcriptome and proteome datasets. **(A)**: Gene and protein differential expression quadrants. The gray dots represent non-differentially expressed genes and proteins. Purple and blue dots indicate genes and proteins that are simultaneously upregulated or downregulated, respectively. Red and green dots denote discordant expression patterns, where either the gene or protein is upregulated or downregulated. Darker colors reflect statistically significant changes, while lighter shades represent non-significant alterations. **(B)**: Venn map illustrating the overlap between differentially expressed genes and differentially expressed proteins. **(C)**: Bubble map of KEGG enrichment analysis. Larger bubbles indicate pathways containing more differentially expressed protein-coding genes. The bubble color scale ranges from blue to red, with smaller p-values (greater significance) represented by redder hues. **(D)**: KGML interaction network diagram. Each node represents a pathway, with node size corresponding to its degree of connectivity and node color denoting its KEGG classification.

To explore the correlation between mRNA and protein expression, an integrated analysis of transcriptomic and proteomic data was conducted. This analysis identified 200 overlapping molecules that were significantly altered between the NC and MC groups (**Figure 4A**), comprising 121 upregulated and 79 downregulated targets (**Figure 4B**).

KEGG pathway analysis was conducted on the 200 overlapping DEGs. The top 20 enriched pathways (*P* < 0.05) are presented in **Figure 4B**. These pathways were primarily involved in key biological processes, including lysosome function, focal adhesion, phagosome formation, regulation of the actin cytoskeleton, ECM-receptor interaction, *PI3K-AKT* signaling, apelin signaling, chemokine signaling, adrenergic signaling in cardiomyocytes, platelet activation, B cell receptor signaling, Fc epsilon RI signaling, protein digestion and absorption, complement and coagulation cascades, human papillomavirus infection, coronavirus disease (COVID-19), Epstein-Barr virus infection, Kaposi sarcoma-associated herpesvirus infection, pertussis, and *Staphylococcus aureus* infection (**Figure 4C**).

Furthermore, KGML (KEGG Markup Language) network analysis was employed to map the relationships among pathways commonly enriched in both DEGs and DEPs. Statistical assessment of these pathway interactions revealed that the *PI3K-AKT* signaling pathway exhibited the strongest associations with the *mTOR* signaling pathway and apoptosis (**Figure 4D**).

### 3.5 Metabolomic analysis of TNF-α transgenic mice

**Figure 5:**
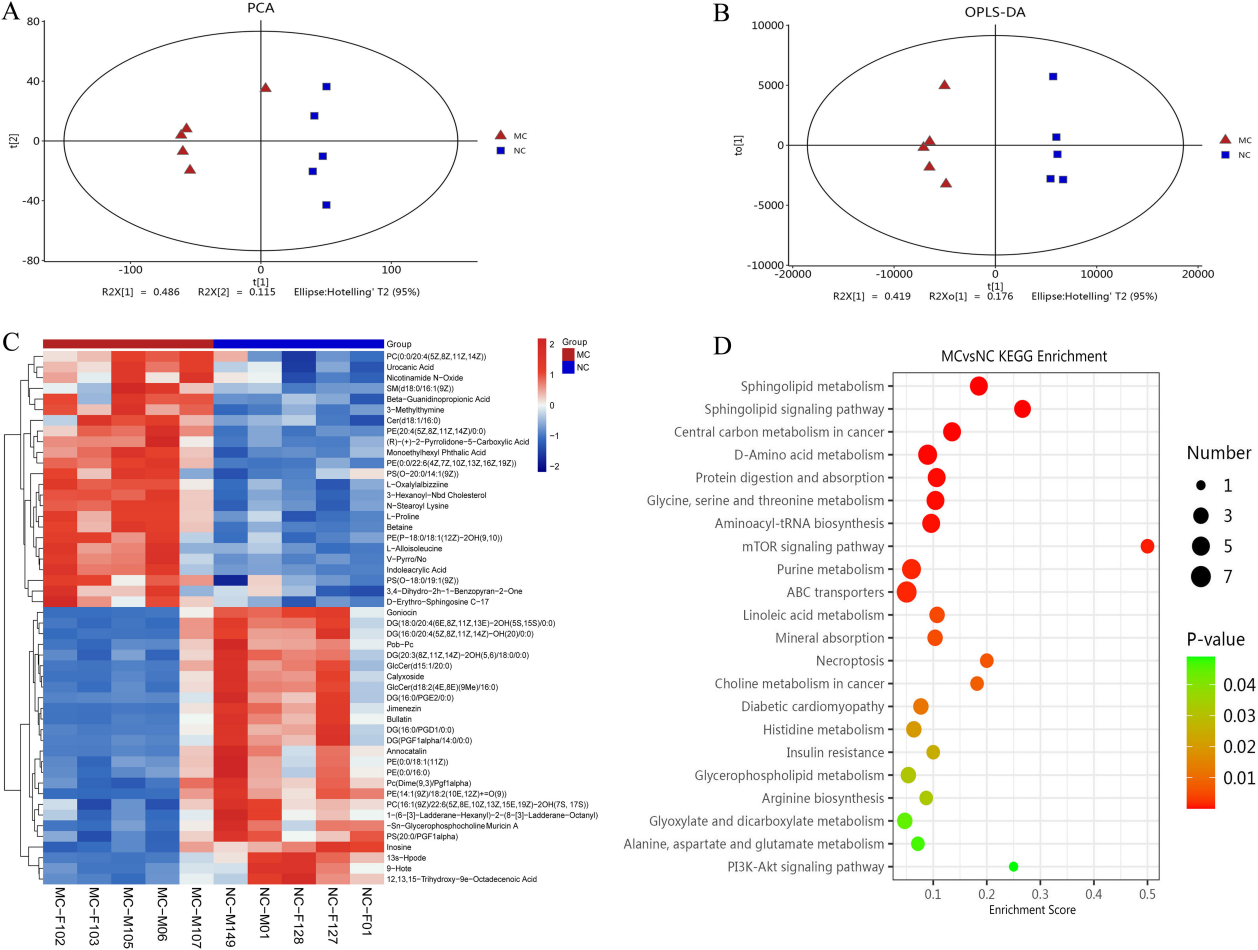
Metabolomic analysis. **(A)**: PCA analysis. **(B)**: Score plots from the OPLS-DA model classifying the MC and NC groups. **(C)**: Clustering heatmap. The color gradient from blue to red indicates increasing expression abundance, with deeper red representing higher abundance. **(D)**: Bubble diagram of KEGG enrichment pathway. Bubble size corresponds to the number of enriched differential metabolites within each pathway. The bubble color gradient from blue to red reflects statistical significance, with smaller *P*-values indicating greater enrichment significance.

Full-spectrum LC-MS metabolomic profiling was conducted to identify metabolites associated with arthritis in TgTC mice. PCA and OPLS-DA revealed distinct metabolic patterns between the MC and NC groups (**Figures 5A, B**). The cumulative R²X, R²Y, and Q² values for the MC and NC groups were 0.723, 0.987, and 0.768, respectively.

Differentially produced metabolites (DPMs) were identified based on a variable importance in projection (VIP) score > 2 and *P* < 0.05. A total of 120 DPMs were significantly altered in the joint synovial tissue of TgTC mice. These metabolites were involved in various metabolic processes, including carboxylic acid and derivative metabolism (n = 12), fatty acyl metabolism (n = 31), glycerophospholipid metabolism (n = 27), organooxygen compound metabolism (n = 7), prenol lipid metabolism (n = 6), sphingolipid metabolism (n = 8), steroid and steroid derivative metabolism (n = 4), and glycerolipid metabolism (n = 3) (**Figure 5C**).

KEGG pathway enrichment analysis of the DPMs identified the top 20 significantly enriched pathways, including the *PI3K-AKT* signaling pathway, alanine, aspartate and glutamate metabolism, glyoxylate and dicarboxylate metabolism, arginine biosynthesis, glycerophospholipid metabolism, insulin resistance, histidine metabolism, diabetic cardiomyopathy, choline metabolism in cancer, necroptosis, mineral absorption, linoleic acid metabolism, ABC transporters, purine metabolism, *mTOR* signaling pathway, aminoacyl-tRNA biosynthesis, glycine, serine and threonine metabolism, protein digestion and absorption, D-amino acid metabolism, central carbon metabolism in cancer, sphingolipid signaling pathway, and sphingolipid metabolism (**Figure 5D**).

### 3.6 Integration of transcriptome, proteome, and metabolome datasets

In this study, several DEGs, DEPs, and DPMs associated with joint synovial inflammation were identified, warranting further analysis of their specific functions. To explore their potential interactions, KEGG pathway enrichment analysis was performed for DEGs, DEPs, and DEMs to identify common signaling pathways.

**Figure 6:**
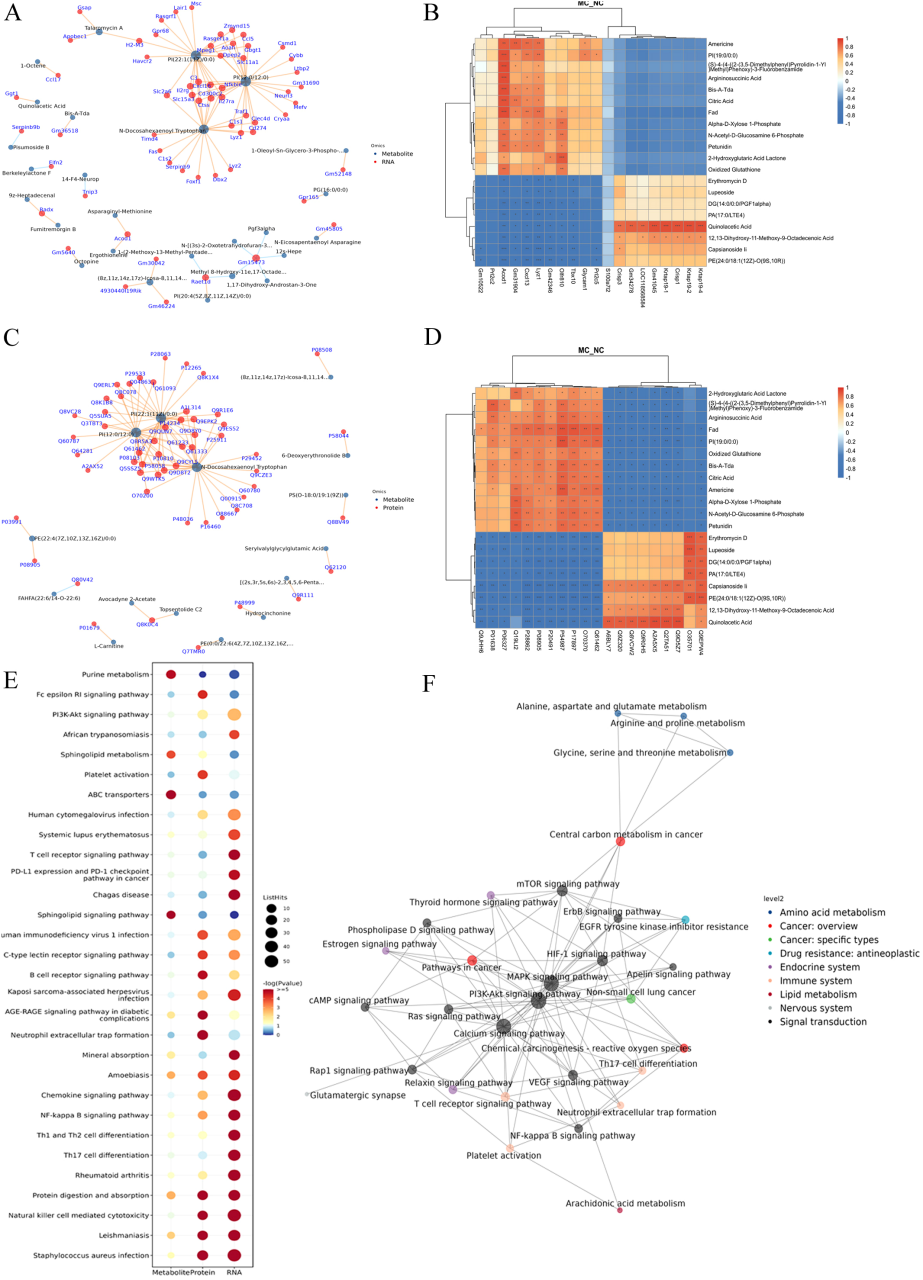
Integration of transcriptome, proteome, and metabolome datasets. **(A)**: Association network diagram of gene-metabolite. **(B)**: The correlation heatmap of the top 20 genes and metabolites. **(C)**: Association network diagram of protein-metabolite. **(D)**: The correlation heatmap of the top 20 proteins and metabolites. **(E)**: KEGG pathway bubble plot of genes-proteins-metabolites. Bubble size indicates the number of differentially expressed components involved in each pathway, while bubble color corresponds to the statistical significance (*P*-value) of pathway enrichment. F: KGML interaction network diagram in DGEs, DGPs, and differential metabolites. The node name is the pathway name, the node size indicates the connection degree, and the node color is identified by the KEGG class of the pathway.

A gene–metabolite correlation network was constructed based on transcriptomic and metabolomic datasets (**Figure 6A**). The top 20 genes (e.g., *Gm10522*, *Prl2c2*, *Acod1*, *Cxcl13*, *Lyz1*, and *S100a7l2*) and top 20 metabolites (e.g., Americine, argininosuccinic acid, citric acid, and N-acetyl-D-glucosamine 6-phosphate) were primarily associated with lipid and glucose metabolism (**Figure 6B**).

A protein-metabolite correlation network was also constructed using proteomics and metabolomics data (**Figure 6C**). The top 20 proteins (e.g., *Myo1f, Jak2, Icam1, and CD14*) and the top 20 metabolites (e.g., 2-hydroxyglutaric acid lactone, argininosuccinic acid, and oxidized glutathione) were also predominantly associated with lipid metabolism and glucose metabolism (**Figure 6D**).

To identify potential biomarkers of joint synovial inflammation in the TgTC mouse model, an integrated analysis of transcriptomic, proteomic, and metabolomic data was conducted. The top five overlapping genes/proteins—*Mpeg1, Enpp2, Tlr2, CD14*, and *Lyz2*—were mainly involved in inflammatory and immune responses, particularly within tryptophan and lipid metabolism pathways (**Table 1**).

**Table 1:** Integrating transcriptome, proteome, and metabolome datasets reveals potentially relevant genes and metabolites.

RNA	Protein	Metabolite	Correlation	P value
*Mpeg1*	A1L314	PI (12:0/12:0)	0.99163764	2.41E-08
*Enpp2*	Q9R1E6	N-Docosahexaenoyl Tryptophan	0.98987162	4.55E-08
*Tlr2*	Q9QUN7	PI (22:1(11Z)/0:0)	0.98871441	7.00E-08
*CD14*	P10810	PI (12:0/12:0)	0.98810852	8.62E-08
*Lyz2*	P08905	PE (22:4(7Z,10Z,13Z,16Z)/0:0)	0.98456163	2.44E-07

Further comprehensive KEGG enrichment analysis of overlapping DEGs, DEPs, and DEMs identified the top 30 pathways, which were predominantly related to immune and inflammatory responses. These included the *PI3K-AKT* signaling pathway, Fc epsilon RI signaling pathway, rheumatoid arthritis, and Th17/Th1/Th2 cell differentiation. Other enriched pathways included NF-κB signaling, chemokine signaling, and sphingolipid metabolism (**Figure 6E**).

Using the KGML network, interactions among overlapping pathways were mapped. The *PI3K-AKT* signaling pathway exhibited the strongest association with the *mTOR* signaling pathway (**Figure 6F**).

### 3.7 Verification of comprehensive analysis results

**Figure 7:**
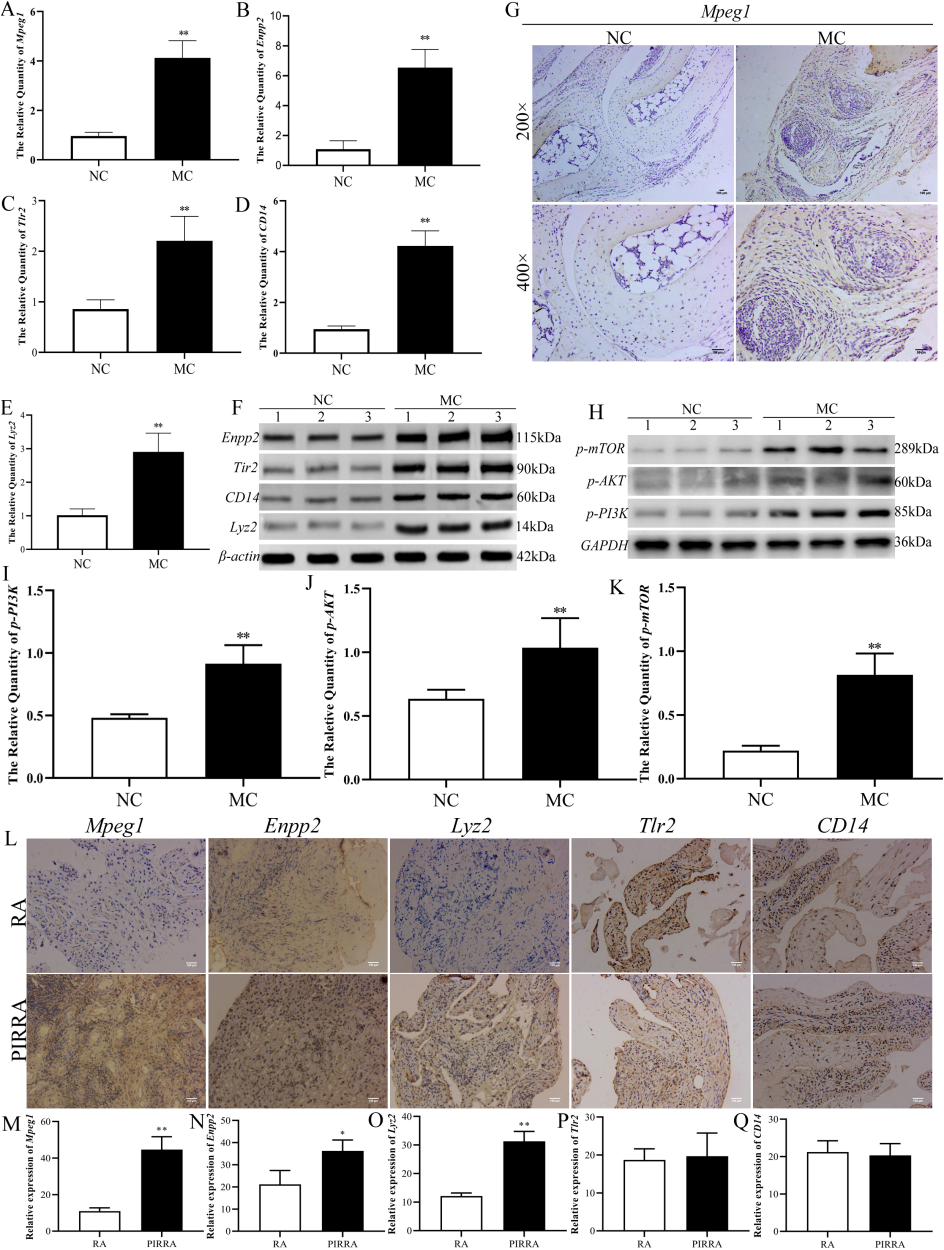
Identification of the target validation. **(A–E)**: Real-time qPCR was used to verify the mRNA expression changes of *Mpeg1, Enpp2, Tlr2, CD14, and Lyz2* in joint synovial tissue of TgTC mice. **(F, H)**: Western blotting verified the changes of *Enpp2, Tlr2, CD14, Lyz2*, and *PI3K-AKT-mTOR* pathway-related proteins in joint synovium tissue of TgTC mice. **(G)**: Immunohistochemistry verified the level of *Mpeg1* protein in joint synovial tissue of TgTC mice. **(I–K)**: the protein expression changes of *p-PI3K, p-AKT*, and *p-mTOR* in joint synovial tissue of TgTC mice. **(L)**: Immunohistochemistry verified the level of *Mpeg1, Enpp2, Lyz2, Tlr2*, and *CD14* protein in the synovial tissue of patients. **(M–Q)**: The relative expression of *Mpeg1, Enpp2, Tlr2, CD14*, and *Lyz2* in synovial tissue of patients. ^*^ *P* < 0.05 or ^**^ *P* < 0.01.

To validate the genes and proteins identified through integrated multi-omics analysis, qPCR, western blotting, and immunohistochemical analyses were performed. As illustrated in **Figures 7A–G**, both mRNA and protein expression levels of *Mpeg1, Enpp2, Tlr2, CD14*, and *Lyz2* were significantly upregulated in the joint synovial tissues of TgTC mice.

Among the top five upregulated genes, *Mpeg1*—processed into active double-stranded molecules in the Golgi apparatus—*Enpp2*, which regulates Golgi positioning to facilitate its biological functions, and *Tlr2* and *CD14*, which form receptor clusters targeting the Golgi apparatus and initiating sustained inflammatory responses, were all closely associated with Golgi apparatus function. Additionally, *Lyz2* exerts its effects via vesicular secretion involving the Golgi apparatus. These observations suggest that chronic inflammation may contribute to Golgi apparatus dysfunction.

To further assess the involvement of the PI3K-AKT-mTOR signaling pathway in the progression of persistent inflammatory arthritis, the protein expression levels of *p-PI3K, p-AKT, and p-mTOR* were evaluated via western blotting. As shown in **Figures 7H-K**, the expression levels of these phosphorylated proteins were significantly elevated in the synovial tissue of TgTC mice.

In addition, expression levels of *Mpeg1, Enpp2, Tlr2, CD14, and Lyz2* were examined in the synovial tissues of patients with rheumatoid arthritis (RA) and persistent inflammatory, recurrent, and relapsing arthritis (PIRRA). Comparative analysis revealed significantly higher expression of *Mpeg1, Enpp2, and Lyz2* in PIRRA synovial tissues relative to RA samples, while no statistically significant differences were observed in *Tlr2 and CD14* expression levels (**Figures 7L–Q**).

These findings suggest that activation of the PI3K-AKT-mTOR pathway, along with increased expression of *Mpeg1, Enpp2, and Lyz2*, may represent core molecular mechanisms underlying the pathogenesis of PIRRA.

## 4 Discussion

PIRRA is a chronic autoimmune disorder characterized by highly heterogeneous and uncontrolled persistent joint inflammation (15). The development of PIRRA involves multiple immune-related factors, including persistent synovitis, smoking, adverse drug reactions, immunogenicity of biologic disease-modifying anti-rheumatic drugs (bDMARDs), recurrent drug side effects, comorbidities, negative disease outcomes (such as secondary fibromyalgia), drug non-compliance, and improper drug use (1). Recent studies suggest that the polarization of SFs and their acquisition of high metabolic activity, inflammation, and invasive phenotypes play a key role in persistent chronic inflammation of RA (16). However, PIRRA remains under-researched, primarily due to its relatively recent recognition as a distinct clinical entity. Consequently, the underlying mechanisms contributing to this “difficult-to-treat” condition are not yet fully elucidated. In the present study, a comprehensive multi-omics approach was employed to investigate the potential molecular mechanisms of PIRRA. The findings suggest that dysregulation of Golgi apparatus function and activation of the PI3K-AKT-mTOR signaling pathway contribute to abnormalities in lipid and tryptophan metabolism, which may be key drivers of PIRRA pathogenesis. Further investigation of these pathways may yield novel insights and support the development of more effective therapeutic strategies for PIRRA.

Multi-omics analysis revealed that *Mpeg1*, *Enpp2*, *Tlr2*, *CD14*, and *Lyz2* were significantly upregulated at both the mRNA and protein levels. These genes are associated with macrophage function, phosphodiesterase activity, toll-like receptors, lipopolysaccharide receptors, and lysozymes. GO and KEGG analyses further indicated that these genes are involved in Golgi apparatus function, immune and inflammatory responses, and the PI3K-AKT-mTOR signaling pathway. Integrated transcriptomic, proteomic, and metabolomic data suggest that these genes are closely associated with disruptions in lipid and tryptophan metabolism. Functionally, they participate in diverse biological processes, including apoptosis, immune response, inflammation, cell invasion, and migration.

Dysfunction of the Golgi apparatus has been implicated in various human diseases, such as neurodegenerative and infectious diseases, cardiovascular conditions, immune system disorders, and cancer (17). Recent studies have demonstrated that the Golgi apparatus plays a critical role in facilitating the effects of platelet membrane–disguised nanoparticles, which can exacerbate joint inflammation and bone erosion (18). Elevated Golgi activity in synovial fibroblasts may contribute to the pathological transition from RA to PIRRA. These findings suggest that Golgi apparatus dysfunction may constitute a novel mechanistic feature of PIRRA pathogenesis.

The PI3K/AKT/mTOR signaling pathway is a core intracellular regulatory axis that plays a crucial role in the abnormal activation and invasion of synovial fibroblasts (SF). Aberrant activation of this pathway promotes the proliferation, migration, and invasion of SF through metabolic reprogramming, ultimately leading to the destruction of articular cartilage and bone. Studies have demonstrated that upregulation of circ_AFF2 markedly increases the expression of phosphorylated PI3K (p-PI3K) and AKT (p-AKT), thereby enhancing the proliferative and invasive properties of SF (19). In an inflammatory and hypoxic microenvironment, elevated expression of hypoxia-inducible factor 1-alpha (HIF-1α) forms a positive feedback loop with the PI3K/AKT/mTOR axis, synergistically driving the metabolic reprogramming of SF (20). Lipid metabolism remodeling mediated by PI3K/AKT/mTOR signaling also contributes significantly to SF invasiveness. For instance, stimulation with tumor necrosis factor-alpha (TNF-α) and platelet-derived growth factor (PDGF) increases the levels of choline kinase and phosphatidylcholine in SFs. Inhibition of choline kinase has been shown to reduce the migratory capacity and anti-apoptotic characteristics of SFs *in vitro*. Moreover, in the K/BxN serum transfer arthritis model, pharmacological inhibition of choline kinase activity substantially alleviated arthritic symptoms (21). Similarly, activation of the sphingosine kinase/sphingosine-1-phosphate (SphK/S1P) axis promotes SF migration and osteoclast activation via stimulation of the PI3K/AKT/mTOR pathway (22). The regulatory role of this pathway also extends to amino acid metabolism. In the inflammatory milieu of the joint, SFs exhibit increased glutamine dependence. This metabolic adaptation provides biosynthetic intermediates and maintains redox homeostasis, supporting the sustained activation and invasiveness of SF (23). Furthermore, the interaction between the PI3K/AKT/mTOR pathway and amino acid metabolism is evident in the regulation of autophagy. Persistent activation of this signaling cascade suppresses autophagy in SFs, leading to uncontrolled proliferation. Conversely, autophagy deficiency results in the accumulation of misfolded proteins, exacerbating endoplasmic reticulum stress and inflammatory responses, thereby promoting the acquisition of an invasive phenotype (24). Notably, aberrant activation of the PI3K/AKT/mTOR pathway may also contribute to drug resistance. Activation of mTORC1 inhibits autophagy by phosphorylating the essential autophagy-initiating protein ULK1, thereby enabling SFs to evade apoptosis and diminishing the therapeutic efficacy of agents such as triptolide (25). In addition, this pathway suppresses ferroptosis by upregulating glutathione peroxidase 4 (GPX4), resulting in reduced sensitivity of SFs to methotrexate (26). It also upregulates drug efflux transporters such as P-glycoprotein, leading to decreased intracellular drug accumulation (27). Taken together, these findings underscore the critical role of the PI3K/AKT/mTOR signaling pathway in regulating SF metabolism, invasiveness, and drug resistance. Elucidating the underlying mechanisms may inform the development of targeted therapies and improve clinical outcomes in PIRRA.

*Mpeg1* was the first gene identified as being highly expressed in macrophages of both humans and rodents (28). The protein encoded by *Mpeg1*, known as perforin-2, is a member of the membrane attack complex/perforin protein (MACPF) family and functions as a pore-forming protein (PFP) within the cholesterol-dependent cytolysin (CDC) superfamily (29). *Mpeg1* is synthesized as an 80 kDa single-chain precursor in the Golgi apparatus, where it is processed into a double-stranded molecule linked by disulfide bonds. This mature form is then packaged into Golgi-derived vesicles and secreted to exert its biological effects (30, 31). Although *Mpeg1* was initially characterized in macrophages, recent findings have demonstrated its expression in fibroblasts as well (32). Under normal physiological conditions, fibroblasts do not express *Mpeg1*; however, pro-inflammatory signals such as TNF-α and lipopolysaccharide can induce its expression. This inducible expression is closely linked to the activation of inflammatory signaling cascades, particularly the PI3K/AKT and NF-κB pathways (33, 34). While *Mpeg1* is known to function as an immune effector and is considered a receptor for apoptotic cells (29), its precise role in various cellular contexts remains incompletely understood. Notably, synovial fibroblasts and macrophages are the two main cell types within the synovial membrane of joints, which suggests that *Mpeg1* may serve as a key biomarker for PIRRA.

*Enpp2* is a member of the extracellular pyrophosphatase/phosphodiesterase (Enpps) family and catalyzes the conversion of lysophosphatidylcholine to lysophosphatidic acid (LPA). The biological functions of *Enpp2* are largely mediated through LPA, which binds to and activates specific LPA receptors. LPA signaling has been shown to influence the spatial positioning of the Golgi apparatus and to promote growth factor–like cellular responses, including proliferation, survival, migration, and cytokine production (35, 36). Several studies have highlighted the role of *Enpp2* in cancer biology, where it affects the migration of liver cancer cells by producing LPA (37). It has also been shown to promote angiogenesis in mice and enhance the mobility of breast cancer cells (38). In inflammatory conditions such as RA, *Enpp2* modulates inflammatory responses by attenuating the effects of interleukins IL-1β and IL-4 in synovial cells (39). Elevated levels of *Enpp2* have been observed in patients with asthma and pulmonary fibrosis (40). Mechanistically, *Enpp2* activates downstream signaling pathways, including Rho, protein kinase C (PKC), and PI3K/AKT, via LPA signaling (41). Activation of the PI3K/AKT axis, in particular, has been shown to enhance cellular migration and invasion, as observed in liver cancer cells (42). Through its capacity to regulate these pathways, *Enpp2* may contribute to the chronic inflammation and invasive characteristics of PIRRA synovium, thereby representing a potential molecular target for further investigation in the context of PIRRA pathogenesis.

*Tlr2* is a member of the Toll-like receptor (Tlr) family and is expressed not only in immune cells but also in fibroblasts (43, 44). *CD14*, a glycoprotein, can either be anchored to the cell membrane via a glycosylphosphatidylinositol (GPI) or secreted in a soluble form (45). As a co-receptor of *Tlr2*, *CD14* plays a crucial role in sensitizing innate immune responses and delivering antigenic substances to various *Tlr* signaling complexes, which in turn trigger a cascade of pro-inflammatory signaling within cells. Upon inflammatory stimulation, *Tlr2* and *CD14* form receptor clusters in the lipid rafts of the cell membrane, which then target the Golgi apparatus, initiating an inflammatory cascade that sustains the inflammatory response (46). Recent studies have demonstrated that *Tlr2* can inhibit proliferation and inflammation in pancreatic cancer cells via the PI3K-AKT signaling pathway (46). Similarly, the formation of a *CD14*-*Tlr4* complex has been shown to activate the PI3K pathway and promote cytokine secretion (47). In RA, both *Tlr2* and *CD14* are highly expressed in synovial fibroblasts, where they contribute to pathological processes including migration, invasion, and cartilage degradation (48, 49). These findings suggest that *Tlr2* and *CD14* serve not only as markers of inflammation but also as potential therapeutic targets in PIRRA, offering insights into the chronicity of the disease.

*Lyz*, also known as cell wall enzyme or N-acetylmuramoyl-hydrolase, is a small monomeric protein that plays a vital role in the innate immune system and is primarily recognized for its antibacterial properties. It is predominantly found in the Golgi apparatus of various cells, where it is secreted via vesicles to exert its biological function (50). Traditionally, lysozyme is known for its antibacterial activity (51), but emerging evidence also suggests that it has immunosuppressive effects (52). For example, lysozyme has been identified as a key regulatory factor in mediating renal fibrosis in diabetic nephropathy, with *Lyz* overexpression promoting the release of glucose-induced fibrosis-related cytokines (53). Interestingly, *Lyz2*, a homolog of lysozyme, is upregulated following activation of *Tlr1/2* and *Tlr4* via the MYD88 pathway. This upregulation is thought to be a secondary response to the accumulation of host-derived lipids in the lysosomal compartment, indicating dysregulated TLR signaling (54). Notably, elevated levels of lysozyme have been detected in the synovial fluid of patients with RA, suggesting a role in the disease’s pathogenesis (55). By extension, *Lyz2* may serve as a potential biomarker and functional contributor to the pathophysiology of PIRRA, particularly through mechanisms involving Golgi apparatus dysfunction and chronic inflammatory signaling.

Through comprehensive analysis of multiple omics datasets, three metabolites—PI (12:0/12:0), PI (22:1 (11Z)/0:0), and N-docosahexaenoyl tryptophan—were identified as key metabolites associated with PIRRA. Among these, PI (12:0/12:0) and PI (22:1 (11Z)/0:0) belong to lipid metabolite molecules, while N-docosahexaenoyl tryptophan is classified as a tryptophan metabolite.

Phosphatidylinositol (PI) is a crucial phospholipid component of eukaryotic cell membranes, essential for maintaining membrane structure and mediating intracellular signaling events (56). Within the Golgi complex, several kinases phosphorylate PI, allowing its conversion into various derivatives, including phosphoinositol (PIP), inositol polyphosphate (IP), complex sphingolipids (IPC), and glycerophosphoinositol (GroPI) (57). These PI derivatives are integral to multiple cellular processes, including cell proliferation, differentiation, apoptosis, metabolism, and membrane transport (58). Notably, abnormalities in PI signaling pathways have been implicated in various human diseases, including genetic retinal degeneration (59) and neurodegenerative disorders (60). Moreover, PI and its derivatives have been demonstrated to play significant roles in sustaining long-term or uncontrolled chronic inflammation (61).

Tryptophan (Trp) is an essential amino acid involved in protein biosynthesis and acts as a precursor for various important bioactive compounds (62–64). Trp metabolism primarily occurs through three pathways: the kynurenine (Kyn) pathway, the 5-hydroxytryptamine (HT) pathway, and the indole pathway. Of these, the kynurenine pathway is most closely associated with immune regulation and inflammatory responses (65). Recent studies have highlighted the increasing importance of tryptophan metabolism in the pathophysiology of rheumatic diseases, particularly in regulating immune and inflammatory functions in conditions such as RA (66). Notably, gut microbiota has been shown to influence tryptophan metabolism, activating aryl hydrocarbon receptors that contribute to the development and progression of rheumatoid arthritis (67). Additionally, metabolites generated via the kynurenine pathway have been shown to activate the PI3K-AKT signaling pathway in tumor colon epithelium, promoting cancer cell proliferation and inhibiting cell apoptosis (68). These findings suggest that dysregulation of lipid metabolism and tryptophan metabolism may contribute to the pathogenesis of PIRRA.

While the present study provides valuable insights into the potential mechanisms underlying persistent inflammatory rheumatoid arthritis (PIRRA), it is not without limitations. First, key findings—such as Golgi apparatus dysfunction, activation of the PI3K-AKT-mTOR signaling pathway, and disruptions in lipid and tryptophan metabolism—require further validation through targeted experimental studies. These mechanistic investigations are planned for future research. Another important limitation lies in the use of the TgTC mouse arthritis model as a substitute for synovial tissue derived from PIRRA patients. Although the TgTC model shares significant pathological features with PIRRA, including joint inflammation, disease progression, and immune dysregulation, interspecies differences constrain its ability to fully replicate the complex pathophysiology of human PIRRA. Human PIRRA is a multifactorial condition shaped by diverse elements such as genetic predisposition, multidrug resistance, immune cell crosstalk, and microenvironmental dysregulation—factors that are not comprehensively modeled in mice. Despite these limitations, the TgTC model remains a valuable experimental platform for investigating the fundamental biological processes involved in PIRRA. It provides a critical foundation upon which future translational and clinical research can be built to advance our understanding and treatment of this difficult-to-treat condition.

## 5 Conclusion

In summary, the comprehensive multi-omics approach integrating transcriptomics, proteomics, and metabolomics has provided valuable insights into the molecular mechanisms underlying PIRRA. The findings highlight Golgi apparatus dysfunction, activation of the PI3K-AKT-mTOR signaling pathway, and disturbances in lipid and tryptophan metabolism as central contributors to PIRRA pathogenesis. Future research focusing on strategies to preserve Golgi function, inhibit PI3K-AKT-mTOR signaling, and restore metabolic balance may offer promising therapeutic opportunities for the prevention and management of this challenging condition.

## Data Availability

The original contributions presented in the study are included in the article/**Supplementary Material**. Further inquiries can be directed to the corresponding authors.
