# Supplementary figures and images for: Integrated multi-omics for potential biomarkers and molecular mechanism of persistent inflammatory refractory rheumatoid arthritis
Source: Front Immunol. 2025 Jul 25;16:1574783. doi: 10.3389/fimmu.2025.1574783 (PMC12331588; doi:10.3389/fimmu.2025.1574783)

# PI3K-Akt signaling pathway(mmu04151)

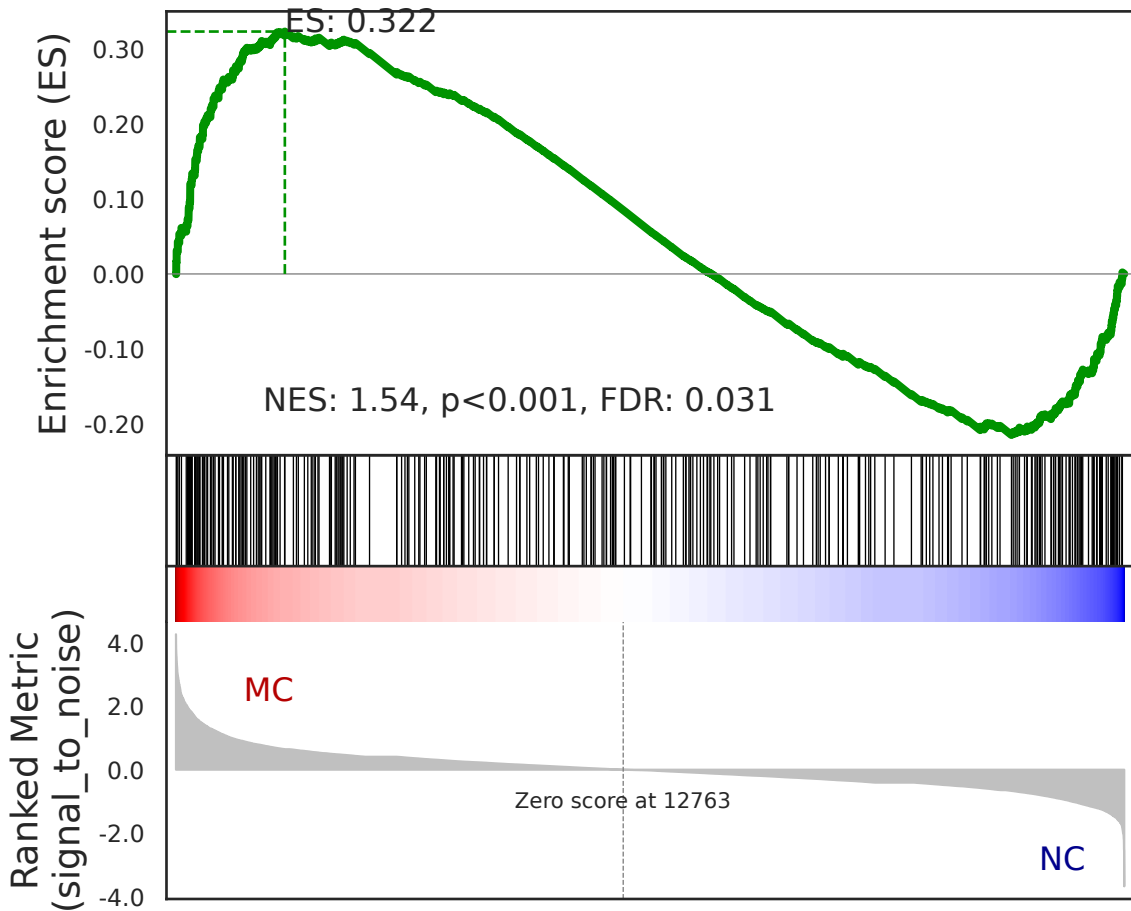

Supplement: Supplementary file 1 [file DataSheet1.pdf]
